# The Influence of Rare Earth Metals on the Microstructure and Mechanical Properties of 220 and 356.1 Alloys for Automotive Industry

**DOI:** 10.3390/ma18050941

**Published:** 2025-02-21

**Authors:** Herbert W. Doty, Shimaa El-Hadad, Ehab Samuel, Agnes M. Samuel, Fawzy H. Samuel

**Affiliations:** 1 Materials Technology, General Motors Global Technology Center, Warren, MI 48092, USA; herb.doty@gm.com; 2 Central Metallurgical Research and Development Institute, Helwan P.O. Box 87, Egypt; shimaam@yahoo.com; 3 Département des Sciences Appliquées, Université du Québec a Chicoutimi, Saguenay, QC G7H 2B1, Canada; ehabfhsamuel@gmail.com (E.S.); agnesmsamuel@gmail.com (A.M.S.)

**Keywords:** aluminum alloys, grain refining, RE, automotive industry, tensile properties

## Abstract

Application of rare earths (RE) as grain refiners is well-known in the technology of aluminum alloys for the automotive industry. In the current study, Al-2.4%Cu-0.4%Mg alloy (coded 220) and Al-7.5%Si-0.35%Mg alloy (coded 356.1), were prepared by melting each alloy in a resistance furnace. Strontium (Sr) was used as a modifier, while titanium boride (TiB_2_) was added as a grain refiner. Measured amounts of Ce and La were added to both alloys (max. 1 wt.%). The alloy melts were poured in a preheated metallic mold. The main part of the study was conducted on tensile testing at room temperature. The results show that although RE would cause grain refining to be about 30–40% through the constitutional undercooling mechanism, grain refining with TiB_2_ would lead to approximately 90% refining (heterogenous nucleation mechanism). The addition of high purity Ce or La (99.9% purity) has no modification effect regardless of the alloy composition or the concentration of RE. Depending on the alloy ductility, the addition of 0.2 wt.%RE has a hardening effect that causes precipitation of RE in the form of dispersoids (300–700 nm). However, this increase vanishes with the decrease in alloy ductility, i.e., with T6 treatment, due to intensive precipitation of ultra-fine coherent Mg_2_Si-phase particles. There is no definite distinction in the behavior of Ce or La in terms of their high affinity to interact with other transition elements in the matrix, particularly Ti, Fe, Cu, and Sr. When the melt was properly degassed using high-purity argon and filtered using a 20 ppi ceramic foam filter, prior to pouring the liquid metal into the mold sprue, no measurable number of RE oxides was observed. In conclusion, the application of RE to aluminum castings would only lead to formation of a significant volume fraction of brittle intermetallics. In Ti-free alloys, identification of Ce- or La-intermetallics is doubtful due to the fairly thin thickness of the precipitated platelets (about 1 µm) and the possibility that most of the reported Al, Si, and other elements make the reported values for RE rather ambiguous.

## 1. Introduction

It has been reported that rare earth metals were used to indicate the lanthanides in the periodic table. This group of elements includes several oxides, mainly scandium (Sc, 21), yttrium (Y, 39), and lanthanum (La, 57), as well as several other elements [1,2,3]. Due to the presence of multiple elements (oxides) in the same ores, the separation of Ce or Le with high purity requires several stages of chemical treatments [4,5,6,7].

Several research publications [8,9,10] have discussed the effectiveness of the application of Ce and Le as grain refiners in the technology of aluminum alloys for automotive and aeronautical applications. The work of Pourbahari et al. [8] shows that the presence of rare earth metals in the form of fine and widely dispersed intermetallics would favor the achievement of finer recrystallized grains during hot deformation by extrusion. Czerwinski [9] analyzed the function of Ce in aluminum melt. His study shows that Ce can act as a grain refiner, while the eutectic modifier can act as a degassing and de-slagging agent, which is caused by its reaction with gas and liquid impurities. Gursoy and Timelli [10] made an extensive analysis of the application of Lanthanide elements in the aluminum–silicon (Al-Si) based alloys. The authors concluded that these elements are ineffective in modifying the eutectic silicon. They can only refine the plate-like morphology at the additions of higher than 1 wt.% of lanthanide. Thus, this behavior is dissimilar to that of Sr and Na, which simultaneously contradicts the current theory of the use of RE as a eutectic modifier similar to Sr [11,12,13].

Hu et al. [14] investigated the role of using Er on microstructure refinement and mechanical properties of die-cast A383 aluminum alloy. Their results indicate that the ultimate tensile strength (UTS) of the used alloy increased significantly with the addition of Er, mainly caused by the refinement of the α-Al dendrites and the eutectic Si, and the reinforcement by the dispersoid Al_3_Er particles. Similar studies were conducted by Jiang et al. [15] using a mixture of La and Ce (up to 0.3 wt%) on the microstructure and tensile properties of A357 alloy. They also arrived at a close conclusion that the addition of RE reduced the sizes of the α-Al primary phase and eutectic Si particles, as well as the SDAS value, and improved the morphology of the eutectic Si particles with significant improvement in the tensile properties. However, the authors did not explore either the mechanisms of the reported observations or the purity of the used RE.

Effects of rare earth elements on microstructure and tensile properties of the Al-Si-Cu alloy at 250 °C have been investigated by Zhang et al. [16]. The authors reported that the addition of 0.2% or 0.4% La significantly enhances the tensile strength of Al-Si Cu alloys, reaching a maximum of 201.48 MPa and 143.91 MPa, respectively. However, the authors did not explain the mechanism behind the claimed improvement. On the other hand, the study conducted by Alkahtani et al. [17] on the A356 alloy (Al-7 wt %Si 0.0.35 wt %Mg) reveals that increasing the amount of La leads to the precipitation of insoluble intermetallics that could negatively affect the alloy performance. Investigations undertaken by Wan et al. [18] on the effect of the addition of RE on microstructures and mechanical properties of an Al-Cu-Mg-Si cast alloy indicate that the mechanical properties of the alloy will reach the highest value when the content of rare earth elements is about 0.7 wt.%.

The present investigations were undertaken to elaborate on the current theories related to grain refining and eutectic Si modification, as well as the combined effect of the concentration of the added RE, coupled with the type of applied heat treatment on the tensile properties of Al-Cu-Mg and Al-Si-Mg alloys using high-purity (99.9%) Ce and La. The results are supported by extensive metallography (employing FESEM and STEM techniques) and tensile testing analysis.

## 2. Experimental Procedures

**Figure 1:**
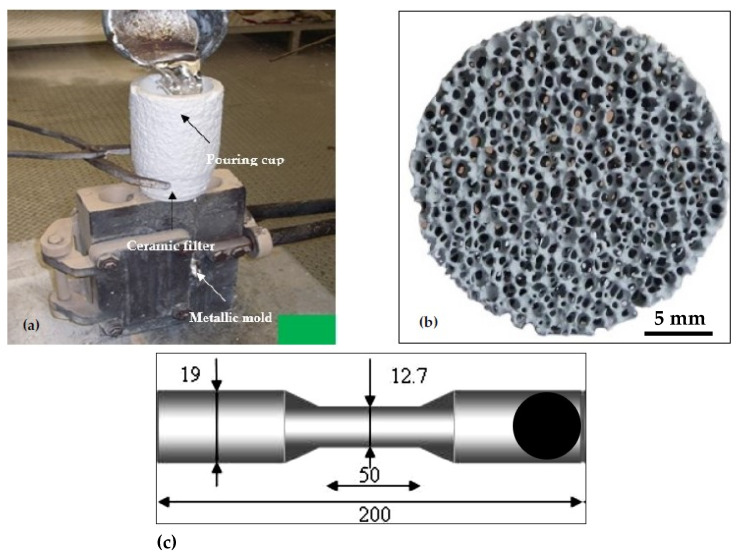
(**a**) Casting set-up used for casting tensile bars; (**b**) 20 ppi ceramic foam filter placed at the bottom of the refractory pouring cup, and (**c**) Dimensions of the ASTM-B108 standard tensile bars produced from the permanent mold. All dimensions are in mm.

Table 1 lists the chemical compositions of the used alloys. To minimize the hot tearing of the 220 alloys during solidification, the alloy was grain refined with TiB_2_ using the Al-5%Ti-1%B master alloy [16,17,18]. The 356.1 alloy was fabricated using A356 alloy as a base, to which Fe and Cu were added using Al-25wt%Fe master alloy and high-purity Cu, respectively. The as-received ingots were melted in a silicon carbide crucible (60 kg capacity) in an electrical furnace. The melting temperature was about 750 ± 5 °C. All alloys were grain refined, using Al-5% Ti-1%B to obtain 0.21 wt.%Ti. The 356.1 alloy was modified with 150 ppm Sr added in the form of Al-10%Sr master alloy. For tensile bars, the required RE levels i.e., 0.2, 0.5, and 1.0 wt.% were made to the alloy melts employing Al-15%La and Al-15%Ce master alloys. In each case, the melts were degassed for 15–20 min using an impeller made of surface-treated graphite through which pure dry argon gas was injected into the molten bath. Thereafter, the melt oxide layers were removed from the melt surface prior to casting. The liquid melt was cast into an ASTM B-108 metallic mold [19] that was preheated at 450 °C to remove moisture—Figure 1. For each melt, samples for chemical analysis were taken prior to casting (at the beginning, the middle, and at the end of the melting process). The chemical analysis was done using a Spectrolab-JrCCD Spark Analyzer (SPECTRO Analytical Instruments Inc., Wilmington, MA, USA).

The applied heat treatments in this study were performed using an air-forced electrical oven. The tensile test bars were solution heat treated (SHT), in addition to T5, T6, and T7 tempering processes. For the T5 treatment, the tensile test bars were directly aged at 180 °C for 8 h, followed by air cooling. For the T6 treatment, the tensile test bars were solutionized at 510 °C/8 h, quenched water (60 °C), and thereafter aged for 8 h at 180 °C. (Tensile bars of 356.1 alloy were kept at room temperature for 24 h for stabilization before aging at 180 °C/8 h.) The parameters of the heat treatments are provided in Table 2.

All tensile specimens (as cast as well as heat treated) in the present study were tested at room temperature using an MTS Servo hydraulic mechanical testing machine (MTS Systems, Eden Prairie, MN, USA) at a strain rate of 4 × 10^−4^ s^−1^. An extensometer (with a 50.8 mm range) was used to measure total elongation before fracture. The tensile parameters, yield strength (YS), ultimate tensile strength (UTS), and % elongation to fracture (%El) were determined from the data acquisition system attached to the machine. In each case, five to eight bars were tested for each alloy/condition.

A Hitachi-SU8000 (Hitachi, Chiyoda, Japan) field-emission scanning electron microscope (FESEM) with an image resolution of 2.1 nm at 1 kV, and 1.5 nm at 15 kV was used in this study. The FESEM is also equipped with a secondary electron detector (SE), a backscatter electron detector (BSE), and an energy-dispersive X-ray spectrometer (EDS) system. Selected samples were examined using transmission electron microscopy, which was used in order to observe, identify, and investigate the coherency of the precipitates with the matrix. The FEI Tecnai G2 F20 (FEI, Lausanne, Switzerland) electron microscope employed is equipped with an EDAX™ chemical analysis system, scanning transmission electron microscopy (STEM), and electron energy-loss spectroscopy (EELS) operating at an accelerating voltage of 200 kV.

## 3. Results and Discussion

### 3.1. Metallurgical Background-Solidification Rate ~0.35 °C/s

**Figure 2:**
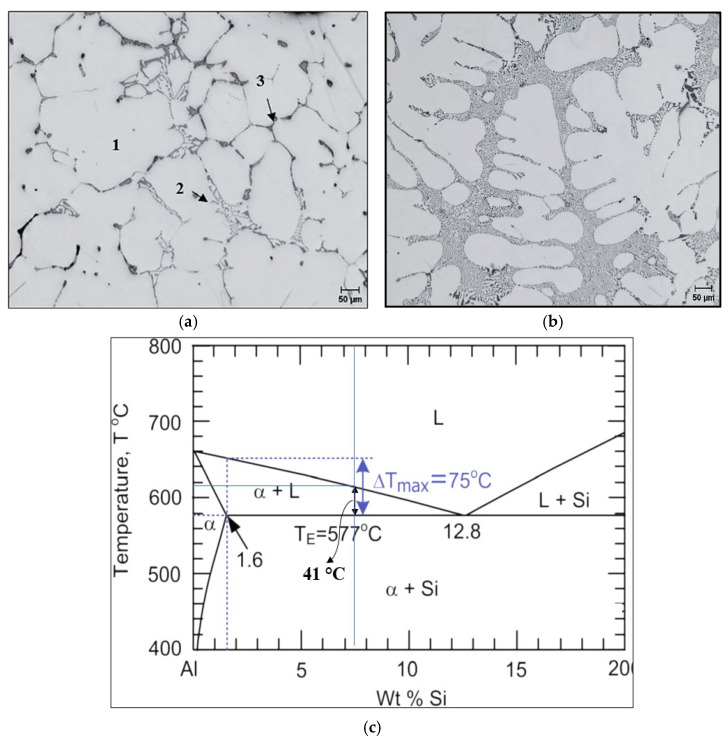
Optical microstructure of as-cast: (**a**) 220 alloy, 1—α-Al, 2—α-AlFeSi, 3—Al_2_Cu, (**b**) 356.1 alloy; and (**c**) Al-Si binary diagram.

Figure 2a,b depict the optical microstructure of the two alloys in the as-cast condition revealing the main microconstituents. The Si content in the 220 alloy is very close to the maximum Si solid solution. According to Soysal [20], an alloy Si content higher than 1.7% would offer good resistance to crack formation, resulting in grain separation since it reflects maximum dT/d(*f_s_*) ^1/2^, where ΔT = 75 °C and *f_s_* is the solid fraction. At *f_s_* = 0.90, dT/d(*f_s_*)^1/2^ = 1432 °C. It is proposed by Kou [21] that lowering the dT/d*f*s will lower the crack susceptibility. Clyne and Davies [22] reported that the separation of dendritic grains or start of solidification cracking will take place at *f_s_* = 0.9. In the case of the 356.1 alloys containing 7.5 wt.%Si (ΔT = 41 °C—see Figure 2c), applying ΔT_max_ results in:

$$\Delta T/d\sqrt{f_{s}}=2\;\Delta T\;\sqrt{f_{s}}/\;(1-\sqrt{f_{s}})=1284\;{}^{\circ}C\;\mathrm{at}\;f_{s}=0.95$$

This is well below the critical condition for cracking at the end of solidification, i.e., 1423 °C. As shown in Figure 2b, the microstructure consists of a well-developed dendritic structure with fine Si particles, filling the interdendritic regions.

**Figure 3:**
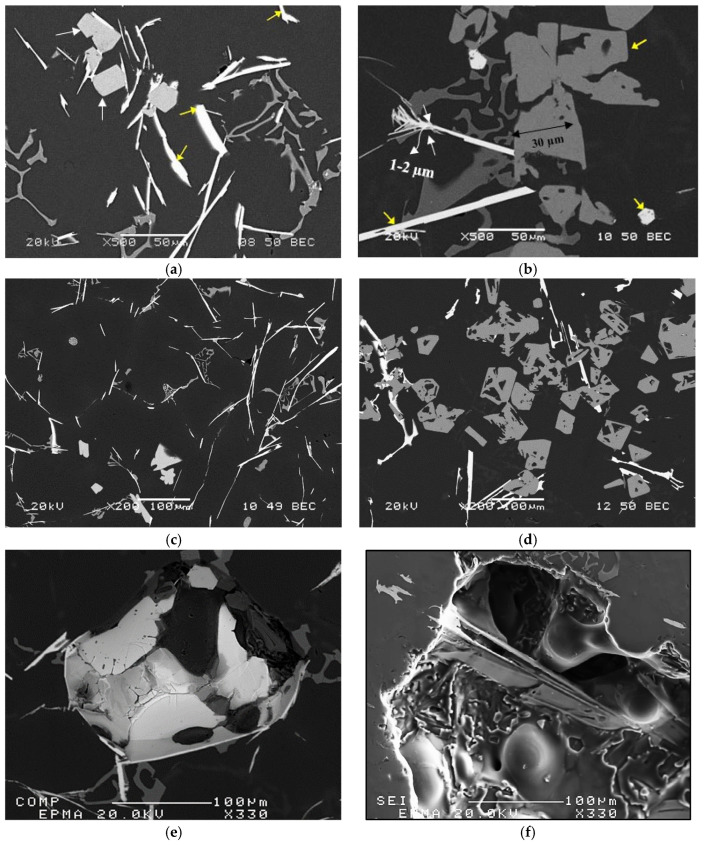

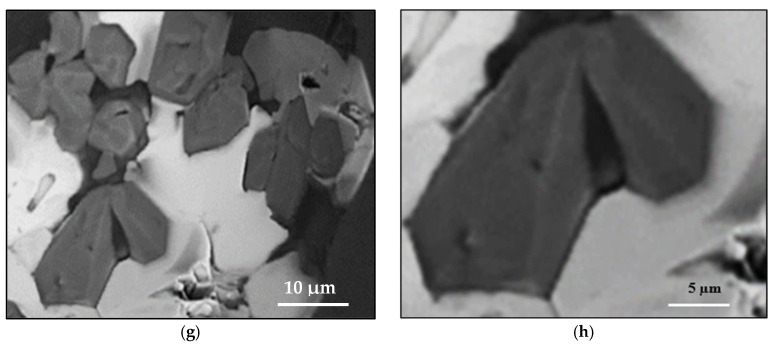
Backscattered electron images of: (**a**) 220 alloy, (**b**) 356.1 alloy, revealing the co-existence of the white phase platelets and the gray-phase massive particles in both alloys-1 wt.%La, (**c**) 356.1 alloy +1 wt.%La-0.06 wt. %Ti, (**d**) 356.1 alloy + 1 wt.%La + 0.27 wt.%Ti, (**e**,**f**) morphology of the white phase in 356.1 alloy containing 1 wt.%La (cavity), (**g**) morphology of the gray-phase particles viewed in a cavity placed on top of a white platelet—note the clear boundaries between the two phases, (**h**) a high magnification image of (**g**) showing the boundaries between the particles of the two phases. White arrows in (**a**) point to the gray-phase particles, whereas yellow arrows point to the white-phase platelets.

Figure 3 exhibits the formation of RE phases in the two alloys grain refined with 0.21%Ti added in the form of the Al-5%Ti-1%B master alloy. In both alloys, the microstructure shows a mixture of thin white platelets (yellow arrows, around 1–2 µm) mixed up with large pieces of gray-phase particles (white arrows, about 100–300 µm). The ratio of the volume fraction of the gray phase to the white one varies from one condition to another depending on the added Ti. To determine the amount of Ti required to convert the white platelets completely into the gray phase, the amount of Ti was gradually increased from 0.06 wt.% (initial level in the as-received ingots-Figure 3c) up to 0.27 wt.%Ti, where only traces of white platelets could be observed (Figure 3d). To elaborate on this point, Figure 3e–g are microstructures viewed from pores, revealing the actual shape and size of both phases. Figure 3h shows the boundary between the two phases.

**Figure 4:**
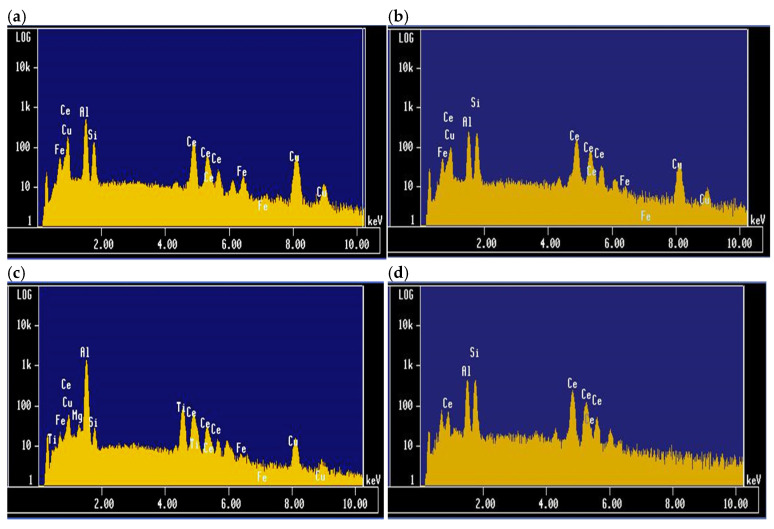
EDS spectra obtained from alloys treated with 1 wt.%Ce; (**a**,**b**) Ti-free 220 alloy, (**c**) 220 alloy treated with 0.21 wt.%Ti, Al/Si ratio > 1, (**d**) 356.1 alloy + 1%Ce, Al/Si ratio~1.

It is clear from Figure 4a,b, corresponding to the white platelets in 220 alloys containing 1 wt.%Ce, that the Al/Si ratios are not constant. In other words, ratios are random due to the fairly thin thickness of these platelets as shown in Figure 3e, which makes it difficult to establish the chemical composition of these platelets with certainty. In contrast, the chemical composition of the gray massive pieces is more accurate due to their large size. In this case, the Si peak seems to have almost vanished—Figure 4c. Figure 4d corresponds to the 356.1 alloy+1wt.% Ce revealing the same peaks as in Figure 4b, except for the absence of the Cu peaks. Although the Al/Si ratios in Figure 4b,d are almost unity, the intensities of the peaks in Figure 4b are slightly lower than those reported in Figure 4d due to the Cu peaks that replace in part the Al and Si in the RE composition.

Table 3 and Table 4 summarize the reported composition of the gray- and white-phase particles observed in 220 alloy +1%La (Table 3) and 356.1 alloy (Table 4). As can be seen, regardless of the alloy composition, the chemistry of the gray phase is consistent, with Al_21_Ti_2_La, or Al_21_Ti_2_Ce in alloys containing Ce. However, all readings obtained from the white phase are ambiguous, which raises questions about the compositions reported in the literature.

**Figure 5:**
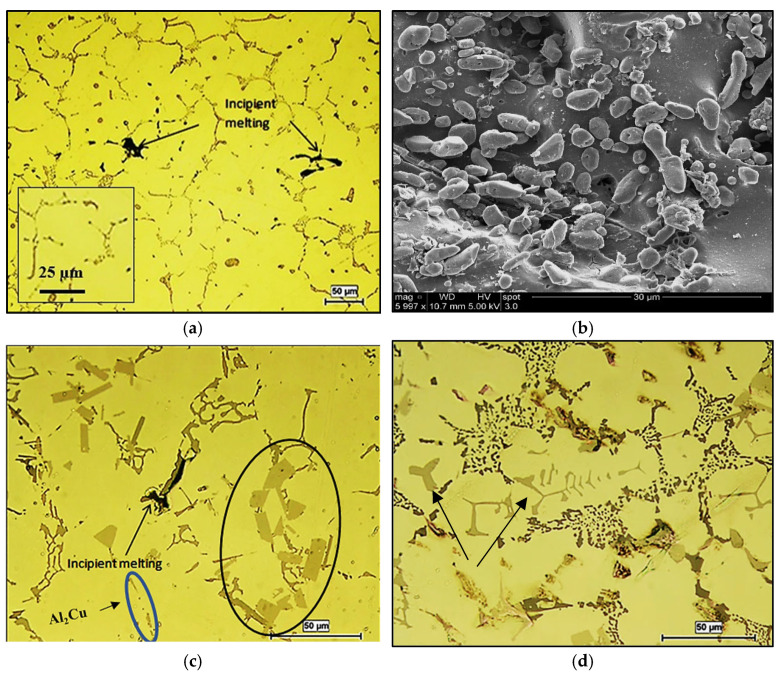
(**a**) Microstructure of the 220 alloy in the SHT condition, (**b**) backscattered electron image of 356.1 alloy—SHT condition, (**c**) 220 alloy +1 wt.%Ce—SHT condition, (**d**) 356.1 alloy in the SHT condition.

**Figure 6:**
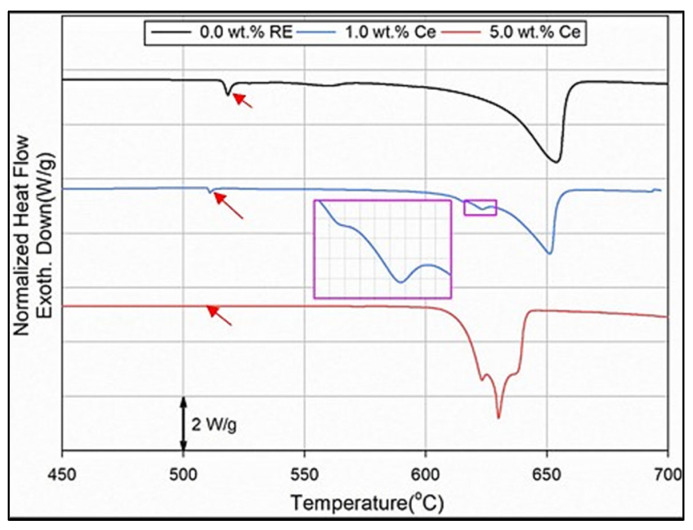
DSC heating curves of 220 alloy containing 0%, 1%, and 5% Ce [24]. The red arrows highlight the disappearance of the Al_2_Cu phase with increase in Ce concentration.

It is known that solutionizing Al-Cu alloys at temperatures close to the melting temperature of the Al_2_Cu phase (normally 500 °C) would cause fusion of this compound, leading to the limitation of the dissolution of alloying elements into the aluminum matrix, hence lowering the alloy strength, and oil leakage [23]. Thus, in the present study, the solutionizing temperature was kept at about 510 °C, which is expected to lead to the dissolution of the Cu phases to strengthen the alloy during T6 and T7 treatments, with minimum dispersed porosity as shown in Figure 5a. The inset in the figure shows few traces of the Cu phase particles, indicating their dissolution with heat treatment. Since the 356.1 alloy was well modified with about 150 ppm Sr, the applied solutionizing treatment resulted in fragmentation of the initially branched fibrous eutectic Si as depicted in Figure 5b. According to the DSC runs performed on 220 alloy with Ce—Figure 6, the peak of the Al_2_Cu phase gradually disappeared during heating with the increase in Ce concentration up to 5%—see red arrows in Figure 6.

In accordance with the observations made by Mahmoud et al. [24,25], Figure 5c exhibits the changes in the microstructure of solutionized 220 alloys with 1 wt.%Ce. As can be seen, the RE phase particles are still visible in the microstructure (black circle), whereas most of the remaining Cu had undergone dissolution (blue circle). Another point to be considered is the change in the phase-formation sequence in Sr-modified Al-Si alloys. Figure 5d displays the formation of α-AlFeSi phase particles prior to the development of α-Al, i.e., higher temperature, explaining its large sized particles (black arrows).

The T6 temper process is very crucial for achieving the required tensile properties of aluminum-based alloys through the precipitation of the dissolved elements in the form of coherent particles. It has been reported that the main hardening agent in Al-Cu alloys is Θ’(Al_2_Cu) in the form of thin rods growing in [010] and [100] directions [26,27], whereas in Al-Si-Mg alloys, hardening occurs due to precipitation of a mixture of GP zones + β’(Mg_x_Si_y_) phases in the form of granular particles [28,29]. The common feature between these precipitates is the interparticle distance which, being very narrow, is capable of restricting dislocation motion and, hence, the need for higher stresses to liberate these dislocations [30]. On going from T6 to the overaging treatment (T7) with the increase in the particles’ size and interparticle distance, the motion of dislocations in the matrix becomes much easier, in addition to the increase in the volume fraction of the soft aluminum matrix [31,32]. 

**Figure 7:**
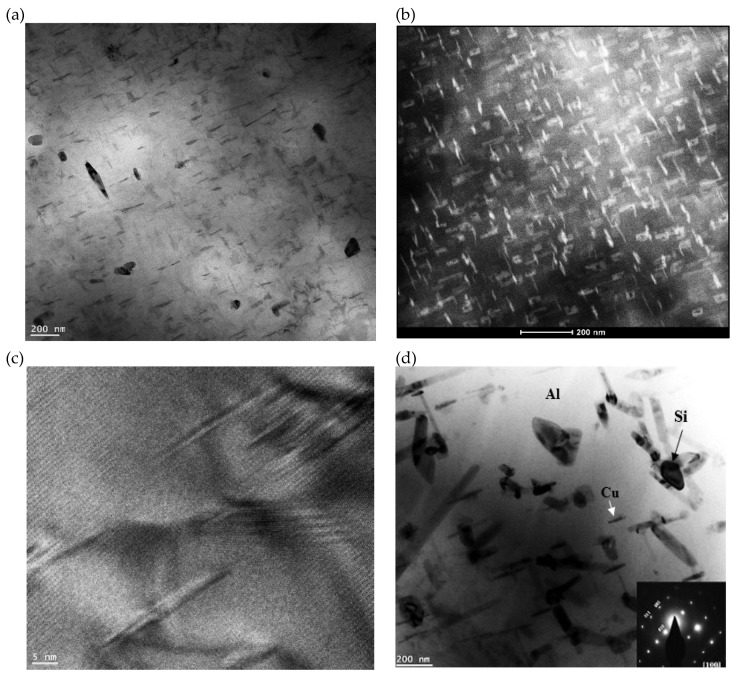
STEM images of 220 alloy: (**a**) T6 condition-bright field, (**b**) same as (**a**) dark field, (**c**) HR image of precipitation in (**a**) showing the continuity of the matrix with precipitates, (**d**) T7 condition.

In the following section, the sequence of precipitation will be examined in the base alloys to avoid interference with the RE. Figure 7 reveals the precipitation behavior in 220 alloy following T6 and T7 treatment.

**Figure 8:**
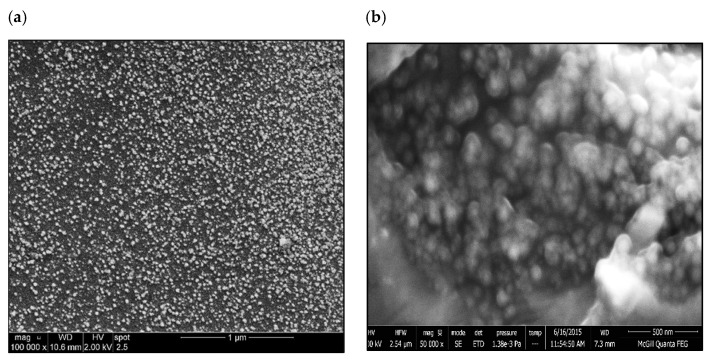
FESEM secondary electron images of 356.1 alloy: (**a**) T6 condition, (**b**) T7 condition.

In the case of 356.1 alloys, the micrographs were taken at an accelerating voltage of 2 kV to be as close as possible to the surface of the polished sample to capture the exact morphology of the precipitated particles. As shown in Figure 8a, dislocations can pass through the particles either by shearing the particles (a shearing mechanism), which depends on the precipitates’ coherency with the matrix, or by looping around them (Orowan looping mechanism) [33]. At an aging temperature as high as 240 °C, the precipitates grow (Figure 8b) to become semi-incoherent or incoherent with the aluminum matrix. In this case, the dislocations can easily bow around the precipitates. Under these conditions, hardening will arise from the bending of the dislocation and the exerting tension stresses of the dislocation according to the Orowan mechanism [34,35].

### 3.2. Tensile Testing Analysis-Solidification Rate About 7 °C/s

**Figure 9:**
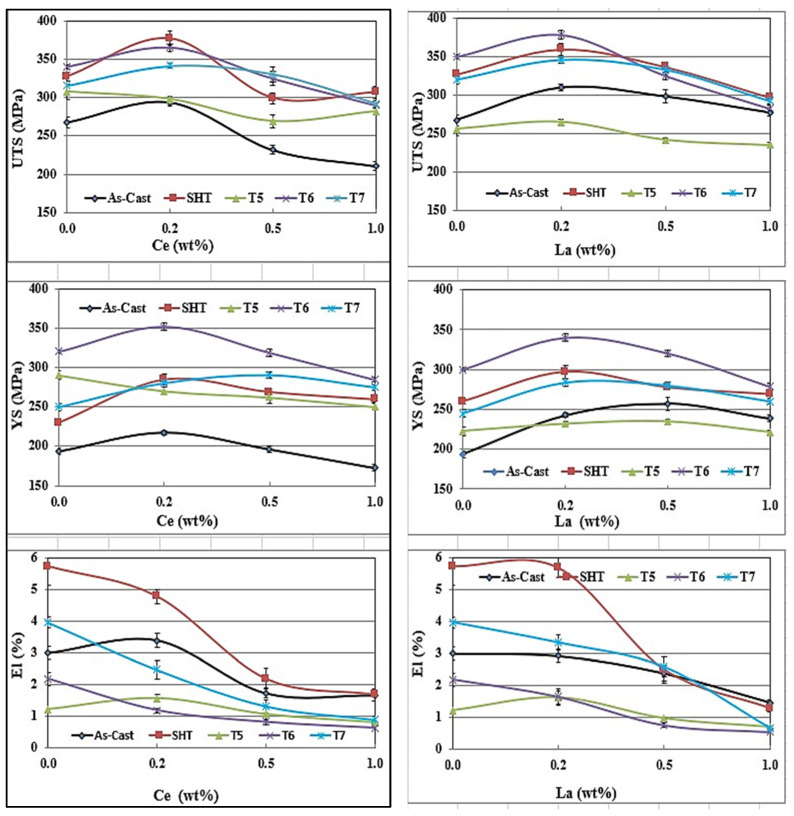
Effect of Ce and La addition on the tensile properties of 220 alloy under different working conditions.

Figure 9 depicts the effect of Ce and La addition on the tensile properties of the 220 alloy under the different applied conditions. Apparently, both Ce and La behave similarly in the sense that the variations are within ±10%. In general, the maximum contribution of RE to UTS and YS values took place after the addition of 0.2%RE, followed by a speedy reduction. Considering the UTS parameter -T6 condition: the addition of 0.2 wt.%RE resulted in an increase of 25 MPa (Ce) and 30 MPa (La). At the maximum addition i.e., 1%, the values of UTS dropped by about 60–70 MPa due to the large volume fraction of insoluble intermetallics. Similarly, the improvement in their levels did not exceed 30 MPa at 0.2% wt. %RE, followed by a reduction of 65 MPa at 1 wt.%RE. It has been found that in conditions with high %El, such as SHT or T7, the impact of the addition of 0.2%RE is noticeable in the range 30–50 MPa. The fact that T5 exhibited minor improvement in the UTS or YS may be interpreted in terms of the size and shape of RE-based intermetallics as displayed in Figure 9. As for elongation to fracture, all working conditions revealed continuous reduction with the increase in the concentration of RE.

**Figure 10:**
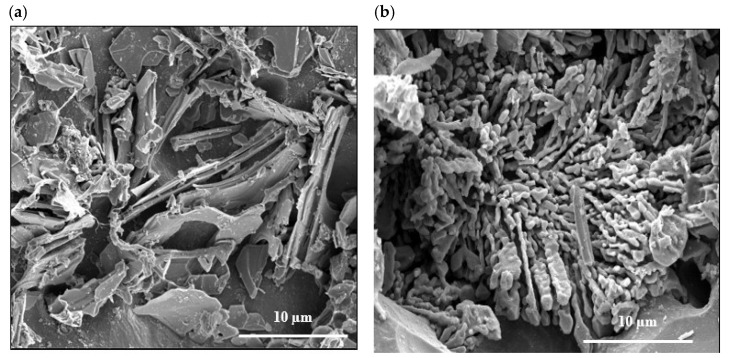

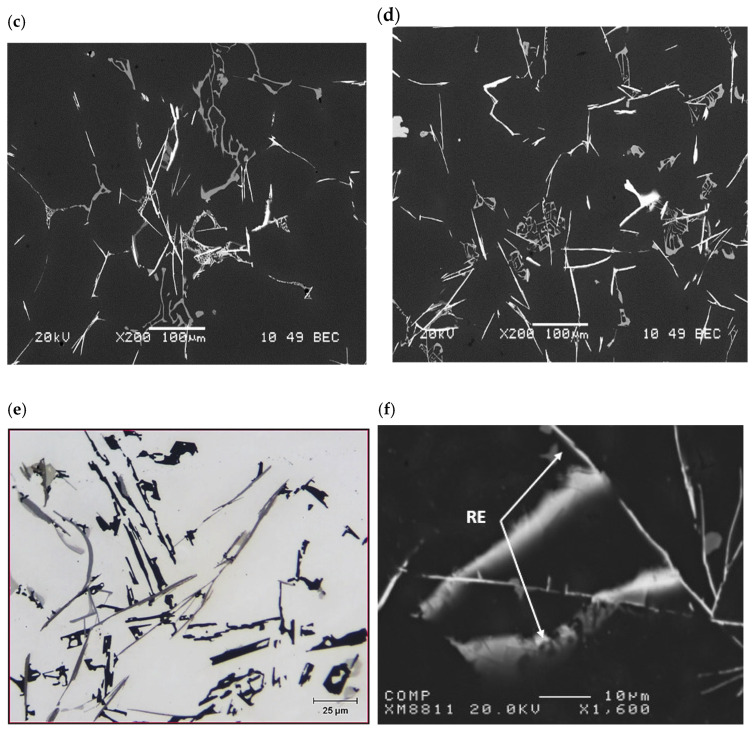
(**a**) Backscattered electron image of deeply etched unmodified 356.1 alloy, (**b**) backscattered electron image of deeply etched modified 356.1 alloy, (**c**) distribution of 0.5% La in the as-cast 356.1 alloy-backscattered electron image, (**d**) 1%La-backscattered electron image, (**e**) 1%La-optical image—note the disappearance of the modified Si eutectic particles, 100 ppm Sr, (**f**) partial fragmentation of RE platelets (1%La) following the solutionizing treatment.

**Figure 11:**
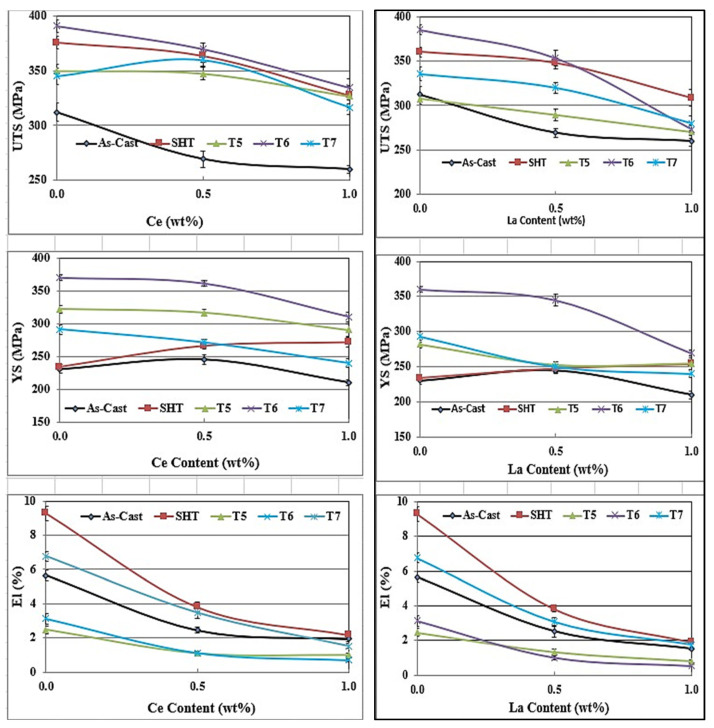
Effect of Ce and La additions on the tensile properties of 356.1 alloy under different working conditions.

Figure 10a reveals the eutectic microstructure of an unmodified 356.1 alloy consisting of long platelets with sharp edges. Once the alloy is modified with 150 ppm Sr, the initial microstructure changes into a fibrous one branching into different directions. The distribution of La in the as-cast condition of the alloy 356.1 is depicted in Figure 10c,d, whereas Figure 10e reveals complete demodification of the eutecic Si particles in the alloy containing less than 100 ppm Sr (which was not the case for the Ce-treated alloys). This leads to the need for increasing the solutionizing time to ensure the complete fragmention of the original Si particles. Figure 10f displays the tendency of some of the La platelets to break into several pieces (white arrows). It should be kept in mind that both 220 and 356.1 alloys were solutionized at 510 °C for 8 h. The effects of Ce and La on the tensile properties of the 356.1 alloy subjected to different working conditions have been demonstrated in Figure 11. Royster [36] has reported that the 300-alloy series is characterized by a better % elongation to fracture compared to the 200 series.

Considering all the factors mentioned above, would explain the main reason as to why the 356.1 alloy lost a major part of its strength, as well as ductility once 0.5%RE had been added, regardless of the applied heat treatment—Figure 11. Analyzing the UTS and YS behavior in both alloys reveals that the reduction in the strength of La-containing 356.1 alloy is greater in the T6 treated condition at 1%La (T6 temper) compared to the loss exhibited by the Ce-treated alloy. The variation in the strength of the alloy in the T6 condition is mainly related to the morphology of the eutectic Si in the Ce- and La-treated alloys.

Thus, generally speaking, the results displayed in Figure 9 and Figure 11 show that for all alloys, regardless of the added amounts of RE, the alloys after the T6 treatment achieved higher (UTS) levels compared to those obtained after SHT-, T5-, and T7-treated conditions. On the other hand, the solution heat-treated alloys revealed ductility values higher than those exhibited by the other conditions. Regarding the effect of RE additions, the results clearly show that the level of RE addition is an effective parameter in the influence of La and Ce on the tensile properties. Alloys with low levels of Ce and La additions exhibit comparatively better UTS values, which tend to decrease with higher additions. The observed decrease in the tensile strength is mainly caused by an increased volume fraction of RE-based intermetallics, particularly at a high concentration of La. Also, the presence of the undissolved intermetallic compounds, coupled with the decrease in the Al_2_Cu precipitates (for the 220 alloys), would reduce the alloy strength.

The difference in properties obtained using the as-received alloys with 0%RE and those after heat treatment, as well as with the addition of various amounts of RE, can be expressed as ΔP, where P = UTS, YS, and %EL, i.e., their contribution (positive or negative) to the as-received alloys. Thus, the ΔP values can be determined as follows:

ΔUTS(x) = UTS(x) − UTS(i)

**Figure 12:**
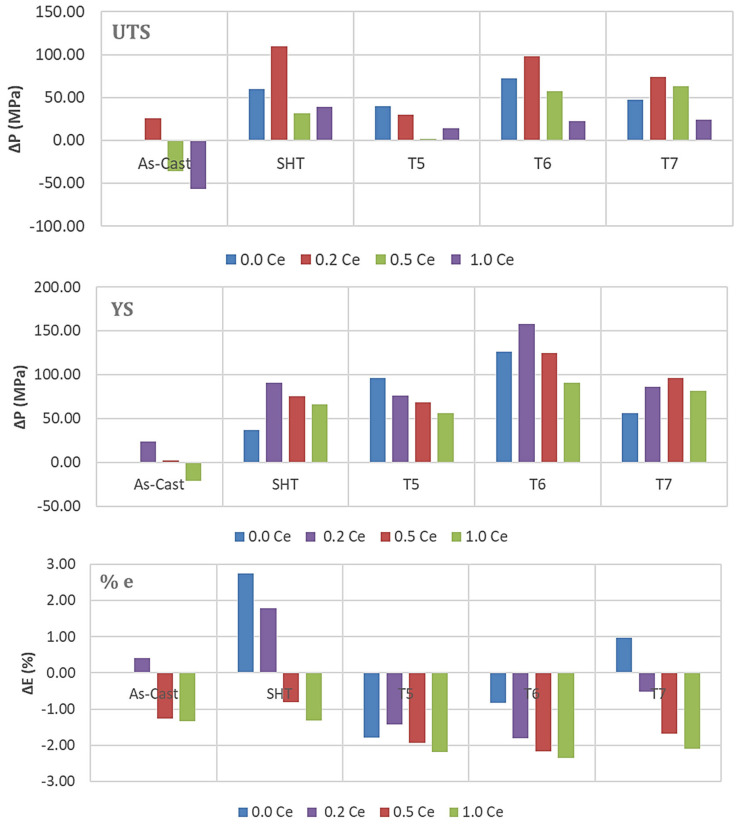
Contribution of heat treatment and Ce addition to the tensile parameters of the 220 alloy; the as-cast sample (without RE) was used as a reference for all the data.

**Figure 13:**
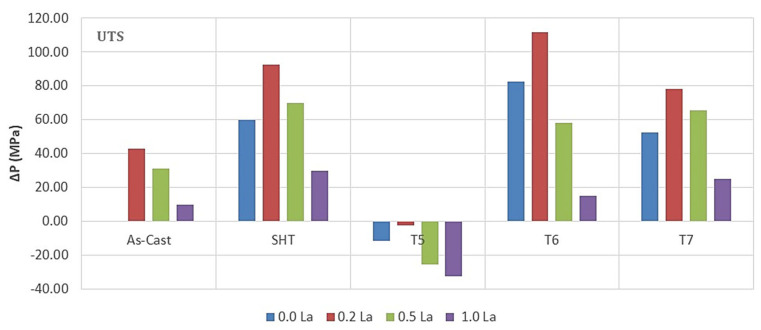

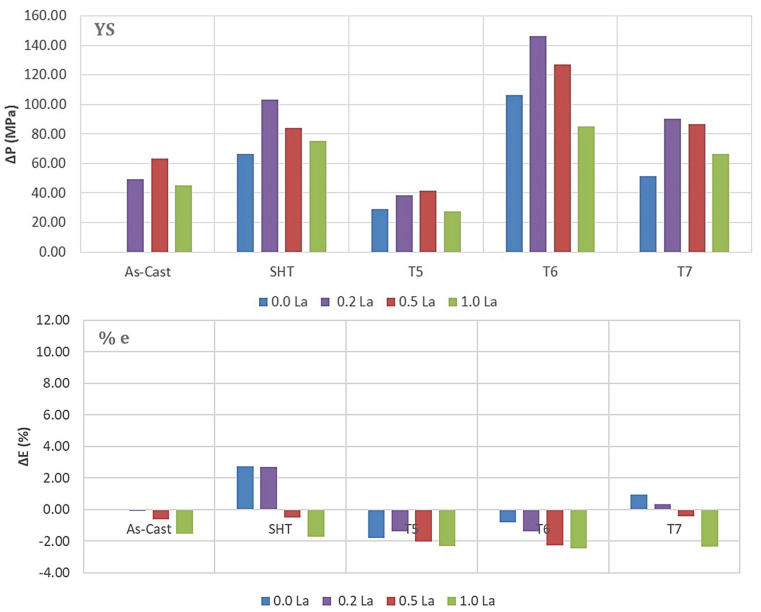
Contribution of heat treatment and La addition to the tensile parameters of the 220 alloy; the as-cast sample (without RE) was used as reference for all the data.

In general, the combined addition of Sr (150 ppm) with 0.2%Re (Ce or La) produced the highest enhancement in the alloy UTS and YS (Figure 12 and Figure 13) in the T6 condition, followed by the T7 treatment. On the other hand, SHT resulted in the best contribution to the alloy ductility. In addition, the results show that substitutional solid solution hardening is more effective than the T5 treatment due to the presence of large amounts of insoluable intermetallics in the as-cast microstructure. However, at higher concentrations of RE, i.e., 0.5%, the initial UTS and YS values were lowered with Ce addition, whereas La offered a somewhat better resistance to softening. Considerable reduction in UTS and YS took place with the addition of 1%RE (Ce or La). Since the increase in strength is associated with a decrease in %elongation to fracture, the ΔP-%El values normally exhibited negative behavior in most cases. Thus, it may be concluded that the best chemical composition to use would be the Sr-modified alloy with 0.2%RE.

**Figure 14:**
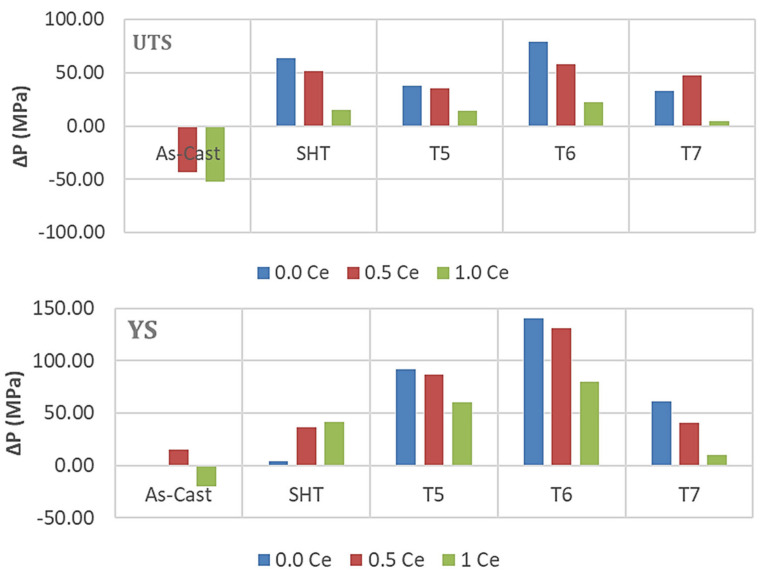

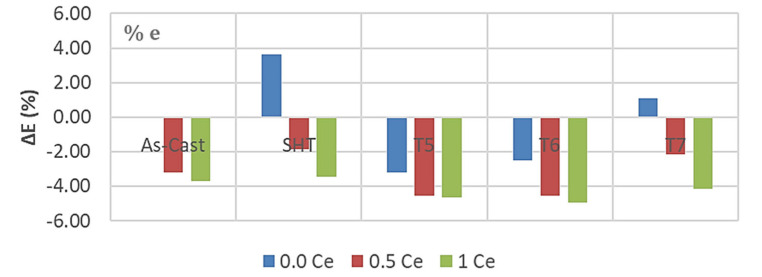
Contribution of heat treatment and Ce addition to the tensile parameters of the 356.1 alloy; the as-cast sample (without RE) was used as a reference for all the data.

**Figure 15:**
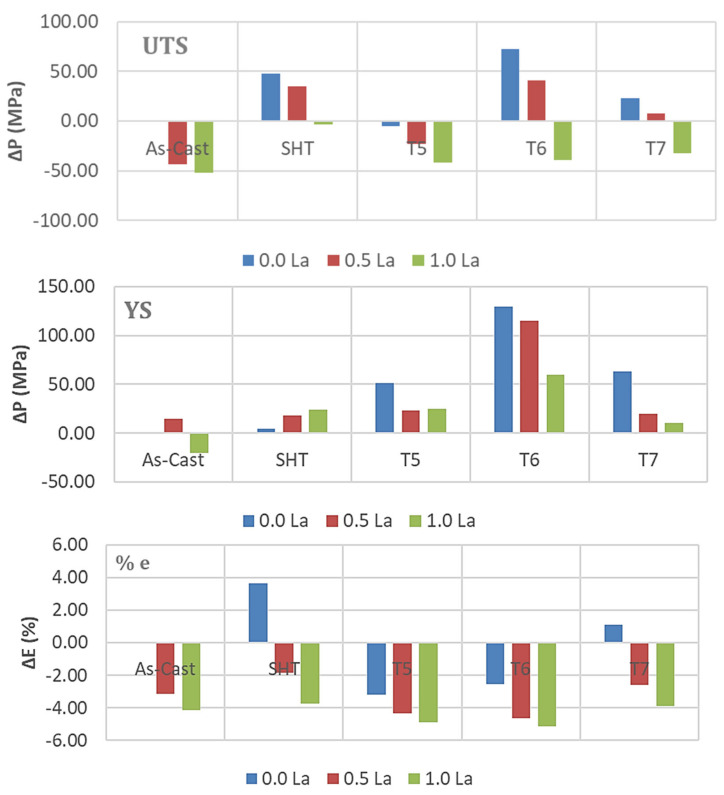
Contribution of heat treatment and La addition to the tensile parameters of the 356.1 alloy; the as-cast sample (without RE) was used as a reference for all the data.

Analyzing the behavior of the tensile parameters of 356.1 alloys—Figure 14 and Figure 15, reveals that for all conditions and RE concentrations, all the as-cast alloys exhibit lower strength levels than the as-received base alloy. Both the UTS and YS exhibited strong responses to the T6 tempering, displaying the highest value for alloys with no RE addition, reaching about 140 MPa and 110 MPa for UTS and YS, respectively. However, the ductility values are all lower than those produced from the 0%RE-base alloy for all conditions.

Thus, for the 356.1 alloys, the plots indicate that the best addition and heat treatment combination for achieving maximum strength would be 0%RE, coupled with T6 treatment. The best ductility value is obtained by the Sr-modified, solution heat-treated 356.1 alloy. Thus, the solution-treated condition would offer the best selection to arrive at a better understanding of the effect of the addition of RE on the ductility of 356.1 alloy.

**Figure 16:**
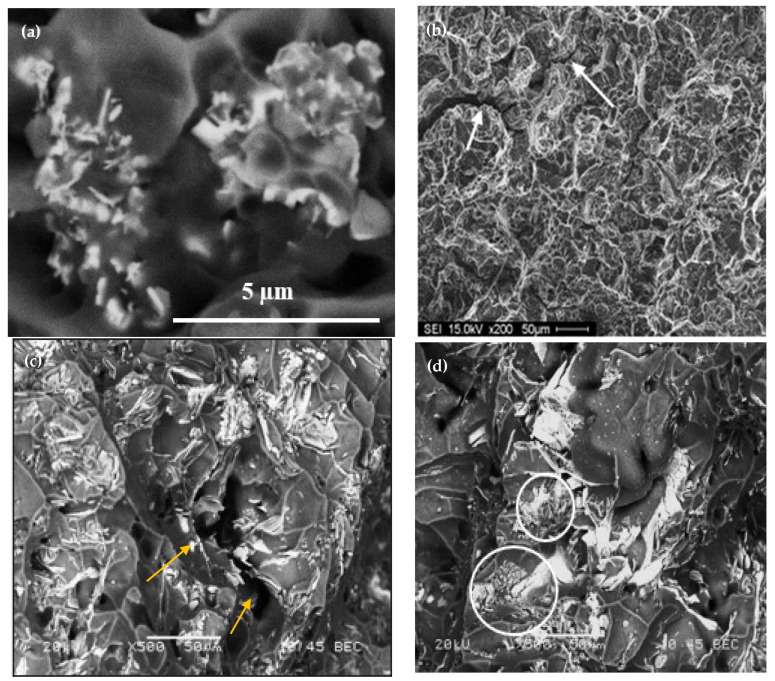
Fracture surface of 356.1 alloy containing La: (**a**) 0.2%La-as cast condition, (**b**) 1%La-as cast, (**c**) 1%La-following T6 treatment, (**d**) 1%La following T7 treatment. The white arrows in (**b**) point to cracks; the yellow arrows in (**c**) point to a secondary crack through the matrix; the circled areas in (**d**) show fractured La-based platelets.

Figure 16a depicts the precipitation of 0.2%La in the form of dispersoids (approximately 300–700 nm), explaining the observed contribution to the alloy strength as mentioned previously. Similar behavior was obtained with the addition of 0.2%Ce. Figure 16b displays the fracture surface of the 356.1 alloy containing 1%La in the as-cast condition, revealing a well-defined network of fine dimple structures. However, deep cracks can easily be observed (white arrows) due to the lack of ductility. The backscattered electron image in Figure 16c exhibits the effect of T6 treatment on the fracture surface of the same alloy, displaying uneven distribution of the La-based platelets and their persistence to the solutionizing treatment (yellow arrows point to a secondary crack passing through the matrix). In the case of T7 tempering (overaging), some of the platelets were fractured (white circles), coupled with an increase in the size of the dimples, contributing to the alloy ductility-Figure 16d.

**Figure 17:**
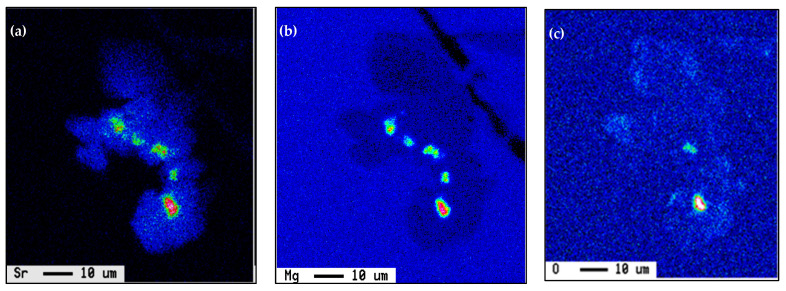
(**a**) Sr, (**b**) Mg, and (**c**) O distribution in a MgSiSr-phase particle, acting as nucleation sites for the formation of La-containing intermetallics.

**Figure 18:**
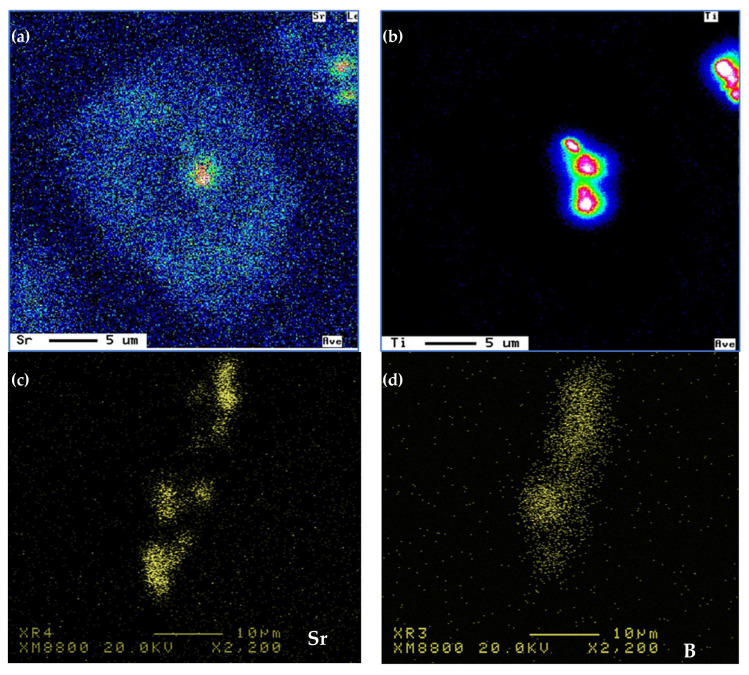

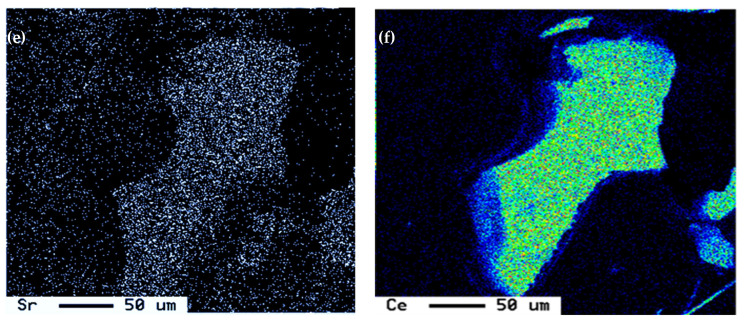
Sr-metal interactions in 356.1 alloy: (**a**,**b**) Sr-Ti, (**c**,**d**) Sr-B, (**e**,**f**) Sr-CE.

Another point to be considered is the Sr-Mg interaction and its effect on the actual free-Mg available for the formation of Mg_2_Si-phase particles during artificial aging, i.e., T6 and T7 treatment. Figure 17 shows the Mg and Sr distribution in a MgSi_2_Sr compound [36]. In addition, Sr reacts simultaneously with other additives, such as Ti, B, and RE as displayed in Figure 18. As a result, the effectiveness of Sr as a Si-modifier agent will be reduced, leading to partial modification as described in Figure 10.

Drouzy et al. [37] proposed the concept of the quality index (*Q*), which can be used to reveal the cast Al alloy’s performance. The quality index can empirically be expressed as follows:

$$Q=S_{UTS}+dlog\left(e_{f}\right)$$

*S_UTS_* refers to UTS (MPa), *e_f_* refers to the %elongation to fracture, and *d* is a constant. For the same alloys, the yield strength (*S_p_*_(*ys*)_) was described as:

$$S_{p(ys)}=aS_{UTS}-blog\left(e_{f}\right)+C$$

where a, b, and c are material constants.

**Figure 19:**
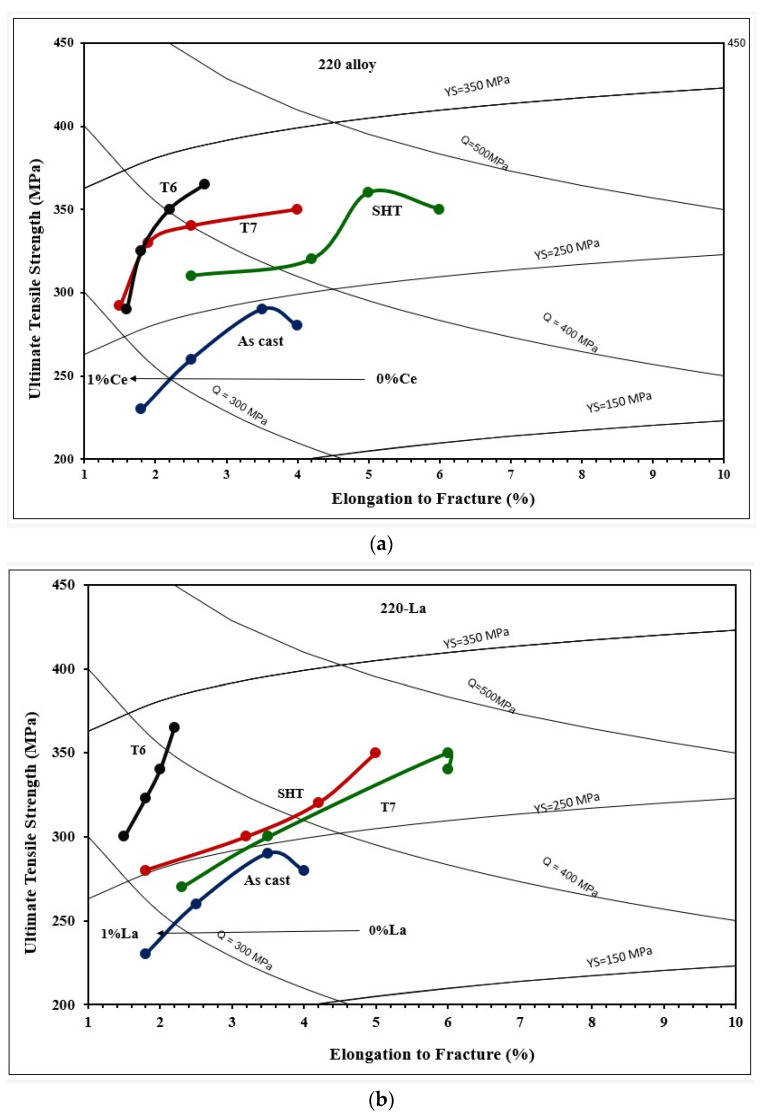
Quality index charts obtained from 220 alloy as a function of applied heat treatment and added RE: (**a**) Ce and (**b**) La. The colored lines in the two plots represent the heat treatment condition, i.e. As-cast, SHT, T6 and T7 The dots on each colored line show the Ce and La additions of 0%, 0.2%, 0.5% and 1%, on going from left to right.

Figure 19 presents the quality charts corresponding to 220 alloys with Ce (Figure 19a) and La (Figure 19b). Since the Q value is the product of UTS and %El, alloy ductility is an important parameter that is affected by the density of the precipitate-hardening particles and amount of added RE, as well as the fraction of RE platelets to compacted particles (sludge) caused by RE–Ti interaction. Thus, it is not expected that a certain amount of Ce should produce the same effect as that could be obtained from the same amount of La, which is clear from comparing Figure 19a with Figure 19b. In general, T6-treated samples possessed significantly higher strength compared to the initial condition (as-cast-0%RE).

Also, the addition of RE has no marked hardening effect on T6-treated alloys. T7-treated samples exhibited the most fluctuation in values on going from Ce to La, which added RE due to variation in %El in each case. As mentioned previously, the 220 alloy was grain refined with TiB_2_ (0.21%Ti) to reduce its susceptibility to hot tearing during casting.

**Figure 20:**
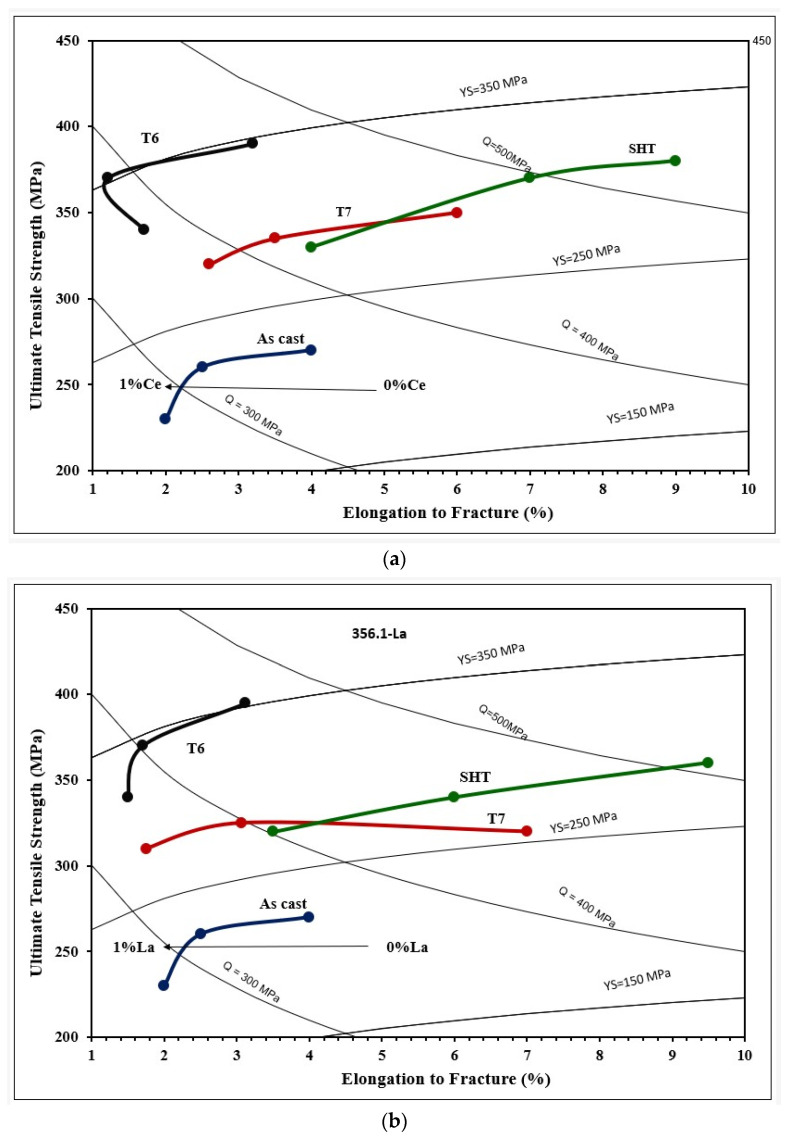
Quality index charts obtained from 356.1 alloy as a function of applied heat-treatment and added RE: (**a**) Ce, (**b**) La. The colored lines in the two plots represent the heat treatment condition, i.e. As-cast, SHT, T6 and T7. The dots on each colored line show the Ce and La additions of 0%, 0.5%, and 1%, on going from right to left.

Figure 20 depicts the Q charts generated from the tensile data presented in Figure 15. The 356.1 alloy is characterized by its high ductility (SHT) caused by fragmentation of the eutectic Si particles, leading to an increase in the volume fraction of α-Al, and high strength, especially after the T6 aging treatment. As shown previously, RE possesses a high affinity to react with Sr, Fe, and Cu, which reduces their actual free concentrations in the alloy and, hence, the alloy tensile properties. As mentioned previously, both Ce and La reflected similar behavior for SHT and T6 conditions. However, the T7-treated samples change their behavior within the same alloy, which mainly occurs due to the variation in %El caused by the above-discussed variables.

## 4. Conclusions

Based on the results documented in the present work, the following highlights may be drawn:

1. The effectiveness of RE as a grain refiner or eutectic Si modifier depends mainly on the purity of the used RE, i.e., free from tramp elements, as well as the concentration of the added RE.
2. RE has a moderate grain refining effect caused by constitutional undercooling. When RE is present in the melt in the form of finely dispersed dispersoids, grain refining takes place as well, by heterogeneous nucleation.
3. When the molten metal is well degassed and filtered prior to pouring into the mold, grain refining due to precipitation on RE oxides is questionable.
4. The addition of 1%La to Sr-modified melt is capable of causing serious demodification if Sr is less than 100 ppm. On the other hand, Ce has no affinity to react with Sr. In other words, REs have no modification effectiveness.
5. The volume fraction of insoluble RE depends on the amount of used Ti due to the formation of RE–Ti compounds.
6. Due to the thickness of RE platelets (less than 1 µm in Ti-free melts), the exact composition of these platelets is random.
7. The addition of 0.2% RE would enhance the alloy strength by about 10–15%. Beyond this concentration, a marked degradation in the alloy strength is likely to take place.
8. Among all the applied heat treatments, T6 represents the source of maximum hardening (over 130 MPa), whereas the solutionizing treatment is the best choice for high ductility.
9. The addition of 0.2 wt.%RE has a hardening effect caused by the precipitation of RE in the form of dispersoids (300–700 nm).

## Figures and Tables

**Table 1:** Chemical analysis of the used alloys (wt.%).

Chemical Analysis (wt%)								
Alloy	Elements							
	Cu	Si	Fe	Mn	Mg	Ti	Zn	Al
220	2.4	1.2	0.4	0.6	0.45	0.21	---	Balance
356.1	0.25	7.25	0.54	0.36	0.45	0.06	0.35	Balance

**Table 2:** Details of the applied heat treatments.

Alloy Type	Condition	Solutionizing Treatment		Quenching Media	Aging Temperature (°C)	Aging Time (h)
		Temp. (°C)	Time (h)			
220/356.1	SHT	510	8	Warm water (60 °C)	none	none
	T5	none	none		180	8
	T6	510	8		180	8
	T7	510	8		240	4 & 50

**Table 3:** Analysis of the EDS spectra taken from 220 alloy +1 wt.%La.

Phase	Composition (wt.%)						
	Al	Ti	Fe	Cu	Si	La	Possible Composition
Gray-1	84.75	7.215	0.014	0.374	0.358	4.343	Al_21_Ti_2_La
Gray-2	84.27	7.317	0.013	0.344	0.496	4.330	Al_21_Ti_2_La
Gray-3	85.22	7.428	0.009	0.258	0.333	4.320	Al_21_Ti_2_La
Gray-5	84.32	7.730	0.012	0.451	0.408	4.384	Al_21_Ti_2_La
White-1	56.46	0.000	2.598	14.97	9.812	15.83	Al_11_La_3_(Cu,Fe)_4_Si_2_
White-3	56.64	0.000	2.942	14.43	10.12	15.61	Al_11_La_3_(Cu,Fe)_4_Si_2_
White-4	46.52	0.000	0.000	3.426	18.30	31.44	Al_5_La_3_Si_2_
White-4	56.14	0.000	1.541	17.57	12.02	11.91	Al_11_La_3_(Cu,Fe)_4_Si_2_

**Table 4:** Analysis of the EDS spectra taken from 220 alloy +1%La.

Phase Color	Composition (wt.%)						
	Al	Ti	Fe	Cu	Si	La	Possible Composition
Gray-1	85.74	6.676	0.013	0.469	0.801	3.815	Al_21_Ti_2_La
Gray-2	85.39	6.543	0.035	0.606	0.919	3.767	Al_21_Ti_2_La
Gray-3	85.48	6.343	0.046	0.707	0.888	3.764	Al_21_Ti_2_La
Gray-4	85.20	6.439	0.013	0.588	1.033	3.832	Al_21_Ti_2_La
White-1	36.57	0.000	0.000	0.290	25.93	35.23	AlLaSi
White-2	34.72	0.000	0.006	0.344	27.82	35.20	AlLaSi
White-3	50.11	0.000	0.000	0.083	26.49	20.95	Al_2_LaSi

## Data Availability

The original contributions presented in the study are included in the article; further inquiries may be directed to the corresponding author.

## References

[1] Ford A., Huston D., Cloutier J., Doublier M., Schofield A., Cheng Y., Beyer E. (2023). A national scale mineral potential assessment for carbonatite-related rare earth element mineral systems in Australia. Ore Geol. Rev..

[2] Zhou B., Li Z., Chen C. (2017). Global potential of rare earth resources and demand for rare earths from clean technologies. Minerals.

[3] Long K.R., van Gosen B.S., Foley N.K., Cordier D. (2010). The Principle Rare Earth Elements Deposits of the United States—A Summary: Of Domestic Deposits and a Global Perspective.

[4] Shen Y., Jiang Y., Qiu X., Zhao S. (2017). Leaching of Light Rare Earth Elements from Sichuan Bastnaesite: A Facile Process to Leach Trivalent Rare Earth Elements Selectively from Tetravalent Cerium. JOM.

[5] Xu D., Shah Z., Cui Y., Jin L., Peng X., Zhang H., Sun G. (2018). Recovery of rare earths from nitric acid leach solutions of phosphate ores using solvent extraction with a new amide extractant (TODGA). Hydrometallurgy.

[6] Liu F., Porvali A., Halli P., Wilson B.P., Lundström M. (2020). Comparison of Different Leaching Media and Their Effect on REEs Recovery from Spent Nd-Fe-B Magnets. JOM.

[7] Echeverry-Vargas L., Ocampo-Carmona L.M. (2022). Recovery of Rare Earth Elements from Mining Tailings: A Case Study for Generating Wealth fromWaste. Minerals.

[8] Pourbahari B., Mirzadeh H., Emamy M. (2018). The Effects of Grain Refinement and Rare Earth Intermetallics on Mechanical Properties of As-Cast and Wrought Magnesium Alloys. J. Mater. Eng. Perform..

[9] Czerwinski F. (2020). Cerium in aluminum alloys. J Mater Sci.

[10] Gursoy O., Timelli G. (2020). Lanthanides: A focused review of eutectic modification in hypoeutectic Al–Si alloys. J. Mater. Res. Technol..

[11] Cheng Z., Yan H., Zhang S., Zou X., Cao C. (2024). Effect of Rare Earth La on Microstructure, Hardness and Corrosion Resistance of A356 Aluminum Alloy. Met. Mater. Int..

[12] (2024). Microstructure evolution and strengthening mechanism of A356/Al-X-Ce(Ti, C) system by inoculation treatment. J. Mater. Res. Technol..

[13] Tsai Y.-C., Chou C.-Y., Jeng R.-R., Lee S.-L., Lin C.-K. (2011). Effect of rare earth elements addition on microstructures and mechanical properties of A356 alloy. Int. J. Cast Met. Res..

[14] Hu X., Jiang F., Ai F., Yan H. (2012). Effects of rare earth Er additions on microstructure development and mechanical properties of diecast ADC12 aluminum alloy. J. Alloys Compd..

[15] Jiang W., Fan Z., Dai Y., Li C. (2014). Effects of rare earth elements addition on microstructures, tensile properties and fractography of A357 alloy. Mater. Sci. Eng. A.

[16] Zhang S., Su Y., Gong W. (2021). Effects of rare earth elements on microstructure and tensile properties of Al-Si-Cu alloy at 250 °C. China Foundry.

[17] Alkahtani S.A., Elgallad E.M., Tash M.M., Samuel A.M., Samuel F.H. (2016). Effect of Rare Earth Metals on the Microstructure of Al-Si Based Alloys. Materials.

[18] Wan W., Han J., LiI W., Wang J. (2006). Study of rare earth element effect on microstructures and mechanical properties of an Al-Cu-Mg-Si cast alloy. Rare Metals.

[19] (2021). Standard Specification for Aluminum-Alloy Permanent Mold Castings.

[20] Soysal T. (2021). A criterion to find crack-resistant aluminium alloys to avoid solidification cracking. Sci. Technol. Weld. Join..

[21] Kou S. (2015). A criterion for cracking during solidification. Acta Mater.

[22] Clyne T.W., Davies G.J. (1981). Influence of composition on solidification cracking susceptibility in binary alloy systems. Br. Foundrym..

[23] Benyoucef M., Clément N., Coujou A., Kostorz G., Calderon H.A., Martin J.L. (1993). Transmission electron microscope in situ deformation of MC2 superalloy at room temperature. Fundamental Aspects of Dislocation Interactions.

[24] Mahmoud M.G., Samuel A.M., Doty H.W., Samuel F.H. (2020). Role of Heat Treatment on the Tensile Properties and Fractography of Al–1.2Si–2.4Cu and Al–8.0Si–2.4Cu Cast Alloys Modified with Ce, La and Sr Addition. Int. J. Met..

[25] Mahmoud M.G., Samuel A.M., Doty H.W., Samuel F.H. (2020). Effect of the Addition of La and Ce on the Solidification Behavior of Al–Cu and Al–Si–Cu Cast Alloys. Int. J. Met. Cast..

[26] Li H., Huang D., Kang W., Liu J., Ou Y., Li D. (2016). Effect of Different Aging Processes on the Microstructure and Mechanical Properties of a Novel Al–Cu–Li Alloy. J. Mater. Sci. Technol..

[27] Khisheh S., Azadi M., Hendoabadi V.Z., Parast M.S.A., Winter G., Seisenbacher B., Gruen F., Khalili K. (2022). Influence of T6 heat-treating and over-ageing on out-of-phase thermo-mechanical fatigue behaviors of Al-Si-Cu alloy. Mater. Today Commun..

[28] Cai Q., Mendis C.L., Chang I.T.H., Fan Z. (2020). Effect of short T6 heat treatment on the microstructure and the mechanical properties of newly developed die-cast Al–Si–Mg–Mn alloys. Mater. Sci. Eng. A.

[29] Lumley R. (2019). The development of high strength and ductility in high-pressure die-cast Al-Si-Mg alloys from secondary sources. JOM.

[30] Sjölander E., Seifeddine S. (2014). Optimization of solution treatment of cast Al-7Si-0.3Mg and Al-8Si-3Cu-0.5Mg alloys. Metall. Mater. Trans..

[31] Kang H.-J., Park J.-Y., Choi Y.-S., Cho D.-H. (2022). Influence of the Solution and Artificial Aging Treatments on the Microstructure and Mechanical Properties of Die-Cast Al–Si–Mg Alloys. Metals.

[32] Moustafa M.A., Samuel F.H., Doty H.W. (2003). Effect of Solution Heat Treatment and Additives on the Microstructure of Al-Si (A413.1) Automotive Alloys. J. Mater. Sci..

[33] Royster D.M. (1969). Tensile Properties and Creep Strength of Three Aluminum Alloys Exposed up to 25,000 Hours at 200’ TO 400’ F (370’ TO 480’ K).

[34] Fracasso F. (2010). Influence of Quench Rate on the Hardness Obtained After Artificial Ageing of an Al-Si-Mg Alloy. Master Thesis.

[35] Colley L.J. (2011). Microstructure-Property Models for Heat Treatment of A356 Aluminum Alloy. Ph.D. Thesis.

[36] Gil-Santos A., Moelans N., Hort N., Van der Biest O. (2016). Identification and description of intermetallic compounds in Mg–Si–Sr cast and heat-treated alloys. J. Alloys Compd..

[37] Drouzy M., Jacob S., Richard M. (1980). Interpretation of Tensile Results by Means of Quality Index and Probable Yield Strength-Application to Al-Si7 Mg Foundry Alloys-France. Int. Cast Met. J..

